# XGB-BIF: An XGBoost-Driven Biomarker Identification Framework for Detecting Cancer Using Human Genomic Data

**DOI:** 10.3390/ijms26125590

**Published:** 2025-06-11

**Authors:** Veena Ghuriani, Jyotsna Talreja Wassan, Priyal Tripathi, Anshika Chauhan

**Affiliations:** Maitreyi College, University of Delhi, New Delhi 110021, India; vghuriani@maitreyi.du.ac.in (V.G.); priyaltripathi2910@gmail.com (P.T.); anshikachauhan.work23@gmail.com (A.C.)

**Keywords:** XGBoost, human genome, feature selection, machine learning, gastric cancer, breast cancer, lung cancer, pathway analysis

## Abstract

The human genome has a profound impact on human health and disease detection. Carcinoma (cancer) is one of the prominent diseases that majorly affect human health and requires the development of different treatment strategies and targeted therapies based on effective disease detection. Therefore, our research aims to identify biomarkers associated with distinct cancer types (gastric, lung, and breast) using machine learning. In the current study, we have analyzed the human genomic data of gastric cancer, breast cancer, and lung cancer patients using XGB-BIF (i.e., XGBoost-Driven Biomarker Identification Framework for detecting cancer). The proposed framework utilizes feature selection via XGBoost (eXtreme Gradient Boosting), which captures feature interactions efficiently and takes care of the non-linear effects in the genomic data. The research progressed by training XGBoost on the full dataset, ranking the features based on the Gain measure (importance), followed by the classification phase, which employed support vector machines (SVM), logistic regression (LR), and random forest (RF) models for classifying cancer-diseased and non-diseased states. To ensure interpretability and transparency, we also applied SHapley Additive exPlanations (SHAP) and Local Interpretable Model-agnostic Explanations (LIME), enabling the identification of high-impact biomarkers contributing to risk stratification. Biomarker significance is discussed primarily via pathway enrichment and by studying survival analysis (Kaplan–Meier curves, Cox regression) for identified biomarkers to strengthen translational value. Our models achieved high predictive performance, with an accuracy of more than 90%, to classify and link genomic data into diseased (cancer) and non-diseased states. Furthermore, we evaluated the models using Cohen’s Kappa statistic, which confirmed strong agreement between predicted and actual risk categories, with Kappa scores ranging from 0.80 to 0.99. Our proposed framework also achieved strong predictions on the METABRIC dataset during external validation, attaining an AUC-ROC of 93%, accuracy of 0.79%, and Kappa of 74%. Through extensive experimentation, XGB-BIF identified the top biomarker genes for different cancer datasets (gastric, lung, and breast). *CBX2*, *CLDN1*, *SDC2*, *PGF*, *FOXS1*, *ADAMTS18*, *POLR1B*, and *PYCR3* were identified as important biomarkers to identify diseased and non-diseased states of gastric cancer; *CAVIN2*, *ADAMTS5*, *SCARA5*, *CD300LG*, and *GIPC2* were identified as important biomarkers for breast cancer; and *CLDN18*, *MYBL2*, *ASPA*, *AQP4*, *FOLR1*, and *SLC39A8* were identified as important biomarkers for lung cancer. XGB-BIF could be utilized for identifying biomarkers of different cancer types using genetic data, which can further help clinicians in developing targeted therapies for cancer patients.

## 1. Introduction

The human genome regulates health maintenance and susceptibility to diseases. Human well-being is very much controlled through the balance of genomic sequences, which directly correlate with essential biological processes [1]. Genetic mutations, epigenetic alterations, external influences, and other such disruptions to genomic equilibrium can lead to the development of various diseases [2]. Understanding the precise structure, function, and regulation of the human genome is essential for gaining insights into the mechanisms that underlie health and disease progression [3]. As such, there has been an increase in genomics studies, highlighting the critical role of genome-wide alterations in the progression of diseases, one of them being cancer. Lung cancer, breast cancer, and gastric cancer significantly contribute to cancer-related mortality [4]. Lung cancer continues to be the leading cause of cancer deaths [5], gastric cancer ranks fourth in the same [6], and breast cancer has been reported to be the most frequently diagnosed malignancy in women [7]. Survival rates continue to remain low, especially in aggressive or late-stage tumors, despite considerable advancements in the field of understanding these malignancies. Genetic predisposition, environmental exposures, and molecular mechanisms, such as alterations in the genome, are very crucial in the pathogenesis of these cancers [6]. Studying these contributing factors is essential to improve diagnostic and therapeutic strategies.

Breast cancer is one of the most studied cancers in terms of genomics, with various genetic and molecular markers playing a critical role in its development, classification, and treatment. Mutations in genes like *BRCA1* and *BRCA2* are well known for significantly increasing the risk of breast cancer, particularly in hereditary cases [8]. Apart from these, gene mutations such as *TP53*, *PIK3CA*, and *HER2* influence tumor progression, aggressiveness, and response to therapy [9]. Lung cancer is a highly heterogeneous disease with significant genetic variations influencing its onset and progression [10]. The two main types, non-small cell lung cancer (*NSCLC*) and small cell lung cancer (*SCLC*), have distinct genomic alterations. *NSCLC*, which accounts for about 85% of lung cancers, frequently exhibits mutations in genes like *EGFR*, *ALK*, *KRAS*, and *TP53*, each of which has therapeutic implications [11]. However, extensive genomic instability and mutations in tumor suppressor genes such as *RB1* and *TP53* are usually linked with *SCLC*. This makes it more aggressive and challenging to treat [12]. Genomic profiling advancements have facilitated the establishment of precision medicine techniques. This allows treatments to be tailored based on the genetic makeup of the tumor and leads to improved patient outcomes [13].

Although there has been progress in comprehending the molecular mechanisms of cancer, it remains difficult to achieve early diagnosis and targeted treatment. Genomic markers have become key tools for advancing the detection of cancer and prognosis so that scientists are able to gain better insights into the genetic causes of these cancers [14]. Nonetheless, deciphering useful genomic markers from large and complicated datasets demands strong computational methods. Machine learning (ML) has turned into a bedrock in resolving this issue. The supervised ML models are well known for classifying diseased and healthy samples based on training using labeled data and classifying unseen, new samples accordingly [15].

The gastric mucosa is associated with microbial signatures in early GC, as Wang et al. identified using a supervised model of RF [16]. The study included unsupervised methods that were employed to see whether proton pump inhibitors induce any meaningful alterations in the gastric tissue microbiota of dyspeptic patients, regardless of previous infections with *H. pylori* [17]. Supervised methods were chosen because they tend to underperform on smaller datasets. However, unsupervised analysis remains strong due to its ability to conduct data quality checks and identify trends within the data, independent of sample knowledge or existing knowledge [18]. Multiple factors associated with tumor initiation, progression, and metastasis influence gastric cancer. *SDC2* and *CLDN1*, which are cell adhesion molecules, regulate tumor adhesion and invasion, facilitating metastasis [19,20].

Persistent infections, such as those caused by Hepatitis C and pathogenic bacteria (Escherichia coli, Salmonella, and Shigella), pitch to gastric carcinogenesis through chronic inflammation and epithelial dysfunction [21]. Epigenetic changes can cause uncontrolled cell proliferation, such as the Polycomb repressive complex (*CBX2*), causing continuous suppression of tumor suppressor genes [22]. Critical signaling pathways such as *MAPK*, *PI3K-Akt*, *Ras*, and *Rap1* (*PGF*) are also important in driving gastric cancer cell proliferation, migration, and apoptosis resistance [23]. Genomic mutations shape cell proliferation, apoptosis, and immune evasion in breast and lung cancers [24]. Mutations in genes such as *BRCA1*, *BRCA2*, and *PIK3CA* interfere with DNA repair and trigger oncogenic signaling pathways in breast cancer. This leads to tumor development [25,26]. The literature suggests that predicting cancer using gene sequences applying machine learning (ML) methods is a promising research direction.

The supervised ML algorithms are known to describe which sample is sick and which sample is healthy, and this information is employed to train the model to classify new incoming data samples. Feature selection is also an important step in getting the most out of ML. It makes algorithms much more effective. The more the feature space, the less the efficiency. Feature selection helps in decreasing the dimensionality of the feature space without interfering with the important data. It eliminates redundant and irrelevant data while also ensuring that the trained model has no impact from the elimination. Different feature selection algorithms are also available, and it is the responsibility of the scientist to determine and estimate properly which algorithm would be suitable for which task [27]. Utilizing feature selection for the ML model improves the efficiency and overall performance of the model.

This study aims to develop a framework for predicting gastric cancer, lung cancer, and breast cancer from gene sequencing data. An ensemble of feature ranking with supervised ML potentially reveals deeper connections between the genome and the prediction of these cancers. By applying the proposed machine learning (ML) techniques, we aim to identify key genes and pathways that are crucial for cancer detection and classification. Our findings could potentially aid in biomarker discovery and targeted therapeutic interventions. The proposed framework aims to contribute to the field of computational oncology by integrating genomic data with ML for more accurate cancer prediction and biomarker discovery.

## 2. Results

### 2.1. Ensemble Performance

This section presents the results obtained from various supervised ML models applied in this research work. Conventional machine learning methods, such as support vector machines (SVM), logistic regression (LR), random forest (RF), and related methods, were traditionally used for detecting useful patterns in human genetic data aiding in cancer detection [18]. However, issues like the high dimensionality of datasets pose a significant challenge to finding relevant genes as biomarkers for different cancer types. Hence, the current research progressed by first choosing an appropriate feature selection method and then applying supervised learning. eXtreme Gradient Boosting (XGB), a tree-based ensemble method, outran all the other algorithms of RF, Variance Threshold, and Mutual Information (as shown in Supplementary Materials) with an accuracy and Kappa > 90% in cancer detection.

The analysis focused exclusively on finding genes identified as biomarkers by the ensemble of XGB and meta-learners of LR, SVM, and RF. The performance of various ensemble models for gastric, breast, and lung cancer classification is summarized in Table 1, Table 2, and Table 3, respectively. The results indicate that feature selection using XGB significantly enhances the classification accuracy and Kappa (agreement statistics) across all cancer types. The comprehensive XGB-BIF framework involved five-fold cross-validation for training distinct cancer types to enhance predictability and reliability. Additionally, the analysis was extended from finding out predictions using the top 10 to 1000 genes. Frequently identified genes were designated as biomarkers for a specific cancer type. To strengthen the methodological justification, we compared XGB-based feature selection with—(i) LASSO (Least Absolute Shrinkage and Selection Operator), a widely used embedded method known for inducing sparsity through L1 regularization [28]. Both methods were applied to the same preprocessed dataset, and the top-ranked features were used to train identical classifiers (RF and SVMs) to assess downstream predictive performance; (ii) RFE (recursive feature elimination), a wrapper-based feature selection method, has also been used that recursively removed the least important features based on a specified estimator (e.g., RF, SVM, LR), aiming to improve model performance and reduce overfitting; (iii) variance thresholding to eliminate genes (features) that show little or no variation across samples as in high-dimensional genomic datasets, such as gene expression microarrays, RNA-seq, or SNP arrays—many features can be uninformative due to low variance [29].

**Figure 1:**
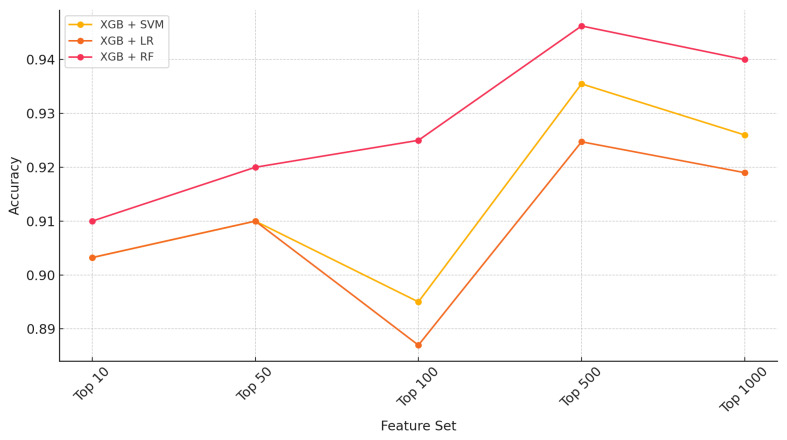
Performance (accuracy) across top n genes (n = 10, 50, 100, 500, 1000) in gastric cancer.

For the gastric cancer use case study (Table 1), the baseline models without feature selection attained the following performance measures—RF performed the best (accuracy = 0.9355, Kappa = 0.8710), followed by LR (accuracy = 0.8817, Kappa = 0.7636) and SVM (accuracy = 0.8387, Kappa = 0.6781). The classification performance significantly improved across all models after applying an XGB-based feature selection with the top n features where n = 10, 50, 100, 500, and 1000 (Figure 1). The ensemble combination XGB + RF achieved the highest accuracy (0.9462) and Kappa score (0.8925), demonstrating the effectiveness of ensemble learning and feature selection (top 500) with the XGB method. LASSO provided the best results with accuracy and Kappa of 0.9234 and 0.8312, respectively. There were moderate improvements for both RF and SVM, though not as impactful as XGB or LASSO. However, XGB-based feature ranking provided the best overall results, especially with RF.

The models for breast cancer classification (Table 2) indicate a good performance with state-of-the-art supervised learners. LR achieved the highest performance without feature selection (accuracy = 0.9864, Kappa = 0.92), while RF and SVM showed comparable results. However, the application of XGB-based feature selection further enhanced performance, with XGB + LR reaching the highest accuracy (0.9918) and Kappa (0.9532). XGB + RF and XGB + SVM also demonstrated improved classification performance, reinforcing the effectiveness of feature selection in further refining model predictions to classify the relevant features for increasing the efficiency of supervised models (Table 2). RFE did not outperform other selectors in this dataset, possibly due to estimator-specific limitations and long computational runtime. Variance thresholding provided competitive results with those of RF (accuracy: 0.9882, Kappa: 0.9268), suggesting that low-variance features in genomic data may contribute little to model discrimination. LASSO provided moderate gains in model interpretability and performance.

**Figure 2:**
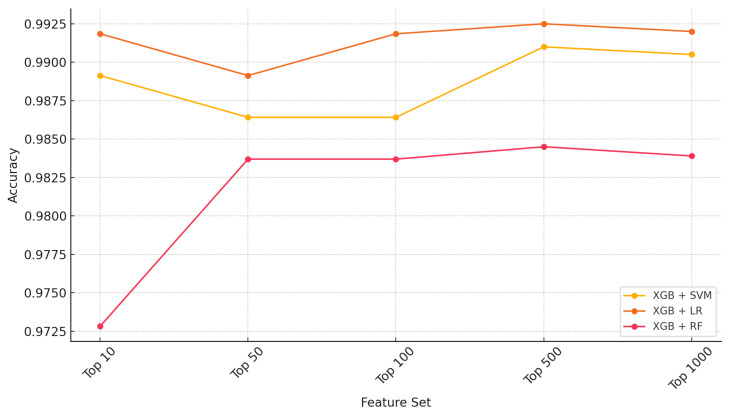
Performance (accuracy) across top n genes (n = 10, 50, 100, 500, 1000) in the breast cancer dataset.

Accuracy improves slightly with more features for all models (SVM, LR, and RF), but the gain plateaus after 500 features (Figure 2). The performance is highest with XGB + LR, indicating a better ability to detect positive (cancerous) cases.

The baseline classifiers performed well in the lung cancer use case (Table 3), with SVM achieving the best initial results (accuracy = 0.971, Kappa = 0.942). XGB-based feature selection significantly improved performance, with XGB + SVM achieving the highest accuracy (0.9941) and Kappa (0.9645), followed by XGB + LR (accuracy = 0.9882, Kappa = 0.9510). Notably, the XGB + RF combination produced a Kappa value of 0.9869, indicating a strong agreement in classification. Feature selection improves classifier performance, especially with more advanced methods like XGB feature ranking. Classifiers achieved superior performance when using the top 500 XGB-ranked features, particularly with SVM, reaching the highest observed accuracy (0.9941) and Cohen’s Kappa (0.9645). In contrast, LASSO combined with SVM or RF yielded slightly lower accuracy (0.9723 and 0.9861, respectively), and RFE-based models showed comparable but not superior results. The variance threshold method underperformed relative to all others.

**Figure 3:**
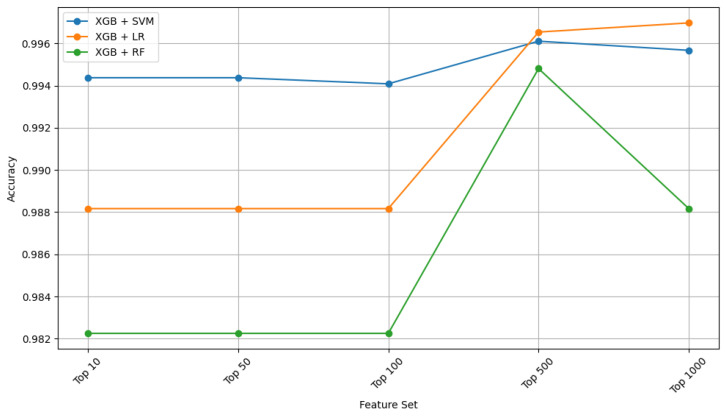
Performance (accuracy) across top n genes (n = 10, 50, 100, 500, 1000) in lung cancer dataset.

XGB + LR/RF/SVM edges out LR/RF/SVM with higher feature counts of top n = 500. Increasing features up to 500 helps, but performance plateaus beyond 500 features (Figure 3).

Comparing all cancer types, feature selection with XGB consistently improved classification performance, with RF and SVM models benefiting the most. The results suggest that combining XGB feature selection with ensemble classifiers, particularly RF and SVM, yields optimal performance in cancer classification tasks. These findings highlight the effectiveness of ensemble learning with XGB-based feature selection in cancer diagnosis, providing a potential framework for improved automated classification in clinical applications. XGB-BIF proved to be the most effective approach for enhancing classification accuracy and agreement.

**Figure 4:**
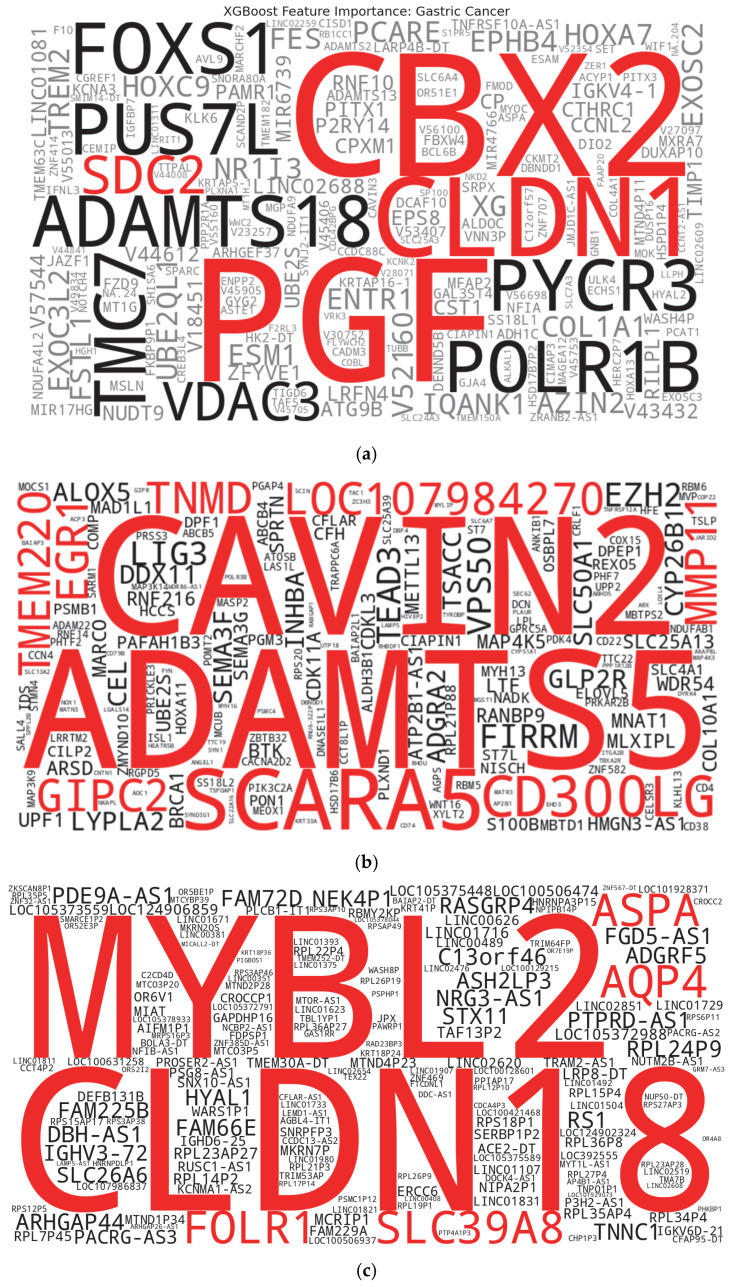
(**a**). Notable biomarkers for the prediction of gastric cancer. (**b**). Notable biomarkers for prediction of breast cancer. (**c**). Notable biomarkers for the prediction of lung cancer.

Another visualization of the top genes (biomarkers) in word cloud form for each cancer type is illustrated in Figure 4a, Figure 4b, and Figure 4c for gastric, breast, and lung cancer, respectively.

### 2.2. External Validation Using the METABRIC Dataset

We also applied the trained models from our primary cancer dataset to the METABRIC (Molecular Taxonomy of Breast Cancer International Consortium) dataset, a large-scale, clinically annotated breast cancer cohort comprising gene expression and clinical data for approximately 2000 patients [30] for external validation. The classification model, originally trained on the [e.g., TCGA] cohort using the top 500 XGB-ranked features, was directly applied to the METABRIC dataset without retraining. We utilized the METABRIC dataset for breast cancer type classification. As shown in Table 4, models that performed well on the internal dataset retained strong predictions on METABRIC, particularly those using XGBoost feature selection combined with SVM (AUC-ROC: 93%, Accuracy: 0.79%, Kappa: 74%). The performance measures are provided in Table 4. These findings support the robustness of our feature selection and classification pipeline, as validating the model on METABRIC provides evidence that the selected features and learned patterns are not overfitted to the original dataset (e.g., TCGA or GEO), and may have broader applicability in clinical settings.

### 2.3. Pathway Analysis

To evaluate the biological relevance of the identified biomarkers, pathway analysis has also been conducted in the current research. This analysis provides knowledge about biological processes linked with the top 10 identified biomarkers offering their potential role in cancer detection. Additionally, Table 5, Table 6 and Table 7 illustrate how several common biomarkers are involved in biological pathways associated with different cancers.

*ADAMTS5* is overexpressed in invasive breast tumors; it promotes extracellular matrix degradation, aiding tumor progression and metastasis [54]. *CAVIN2* is downregulated in breast cancer; loss is linked to tumor progression and metastasis, as indicated in [55]. The loss of *CAVIN2* expression disrupts membrane integrity and has been linked to increased cellular invasiveness, highlighting its function as a tumor suppressor. *MMP11*, another matrix metalloproteinase, is highly expressed in the tumor stroma of breast cancer and plays a key role in promoting tumor invasion and metastasis through the remodeling of the extracellular matrix [56]. Its expression is often elevated in aggressive breast cancer subtypes. *SCARA5* is downregulated in breast cancer and acts as a tumor suppressor by regulating metabolism and immune responses [57]. Its reduced expression has been associated with increased tumor growth and progression. *EGR1* is involved in growth and differentiation and plays a dual role in cancer biology. While it can act as a tumor suppressor in some contexts by inhibiting proliferation and inducing apoptosis, in others, it may function as an oncogene, depending on the tumor microenvironment [58]. These findings not only corroborate the relevance of our feature selection but also highlight potential biomarkers and therapeutic targets in breast cancer.

**Table 6:** Pathway enrichment analysis performed on top-identified genes in the breast cancer dataset.

EntrezID	Gene	Pathway	Role in Breast Cancer
11096	*ADAMTS5*	Extracellular matrix disassembly	Degrades ECM, aiding tumor invasion and metastasis [59].
8436	*CAVIN2*	Plasma membrane tubulation	Plays a role in membrane remodeling linked to cancer progression [60].
4320	*MMP11*	Extracellular matrix disassembly	Breaks down ECM, promoting metastasis in breast cancer [61].
286133	*SCARA5*	Iron ion transmembrane transport	Tumor suppressor regulates iron homeostasis and oxidative stress [62,63].
1958	*EGR1*	Temperature homeostasis	Tumor suppressor regulates apoptosis and cell cycle [64,65].
		Cell proliferation involved in metanephros development	Involved in breast cancer cell growth and proliferation [66].
		Regulation of glomerular mesangial cell proliferation	Regulates tumor microenvironment and immune response [67].
		Positive regulation of gene expression via chromosomal CpG island demethylation	Epigenetic regulation, affecting tumor suppressor activation [68].
		Positive regulation of hormone metabolic process	Influences hormone-driven breast cancer via estrogen receptor signaling [69].

**Table 7:** Enrichment analysis performed on top-identified genes in lung cancer dataset.

EntrezID	Gene	Pathway	Role in Lung Cancer
361	*AQP4*	Vasopressin-regulated water reabsorption	Regulates water homeostasis; implicated in lung cancer metastasis and brain edema [70].
		Bile secretion	Regulates water homeostasis; implicated in lung cancer metastasis and brain edema [70].
443	*ASPA*	Histidine metabolism	May play a role in metabolic reprogramming in lung cancer cells [71]
		Alanine, aspartate and glutamate metabolism	May play a role in metabolic reprogramming in lung cancer cells [71].
51208	*CLDN18*	Virion-Hepatitis viruses	Tight junction protein; associated with lung adenocarcinoma and gastric tumors [72].
		Leukocyte transendothelial migration	Tight junction protein; associated with lung adenocarcinoma and gastric tumors [72].
		Hepatitis C	Tight junction protein; associated with lung adenocarcinoma and gastric tumors [72].
		Cell adhesion molecules	Tight junction protein; associated with lung adenocarcinoma and gastric tumors [73].
		Tight junction	Tight junction protein; associated with lung adenocarcinoma and gastric tumors [73].
		Pathogenic Escherichia coli infection	Tight junction protein; associated with lung adenocarcinoma and gastric tumors [74].
2348	*FOLR1*	Antifolate resistance	Overexpressed in lung cancer; involved in folate metabolism, supporting tumor growth [75].
		Folate transport and metabolism	Overexpressed in lung cancer; involved in folate metabolism, supporting tumor growth [76].
		Endocytosis	Overexpressed in lung cancer; involved in folate metabolism, supporting tumor growth [77].
4605	*MYBL2*	Cellular senescence	Drives cell cycle progression; upregulated in lung cancer promoting proliferation [78].
64116	*SLC39A8*	Ferroptosis	Involved in metal ion transport; linked to altered zinc homeostasis in lung tumors [79].
		Parkinson disease	Involved in metal ion transport; linked to altered zinc homeostasis in lung tumors [79].
		Alzheimer disease	Involved in metal ion transport; linked to altered zinc homeostasis in lung tumors [79].

Studies [80,81] suggest that altered *AQP4* levels may influence tumor cell migration and are associated with lung cancer prognosis. *ASPA* (Aspartoacylase) has recently gained attention for its reduced expression in lung adenocarcinoma, potentially reflecting metabolic dysregulation within tumors. *CLDN18* (Claudin 18), a tight junction protein, has emerged as a particularly relevant biomarker in lung cancer [82]. The *CLDN18* is also being actively targeted in clinical trials for gastric and lung cancers, possibly due to its overexpression in tumors, offering opportunities for targeted therapy [83]. *FOLR1* (Folate Receptor Alpha) is overexpressed in a subset of lung adenocarcinomas, supporting tumor cell proliferation through folate uptake [84]. It has been explored as a therapeutic target using antibody-drug conjugates and folate-linked imaging agents. *MYBL2* (v-myb avian myeloblastosis viral oncogene homolog-like 2) functions as a transcription factor regulating cell progression and is consistently overexpressed in aggressive forms of lung cancer [85]. Its expression correlates with poor prognosis and resistance to certain therapies, positioning it as both a biomarker and a potential therapeutic target. Lastly, *SLC39A8* (Solute Carrier Family 39 Member 8), a zinc transporter, is dysregulated in lung cancer and may influence tumor behavior by breaking homeostasis. Some studies suggest that its altered expression may be linked to immune modulation and chemotherapy response, supporting its exploration as a prognostic biomarker [86]. Together, these genes reflect key biological pathways involved in lung cancer and represent promising candidates for biomarker development and targeted intervention.

### 2.4. Explainable AI Techniques to Demonstrate the Influence of Individual Genes

**Figure 5:**
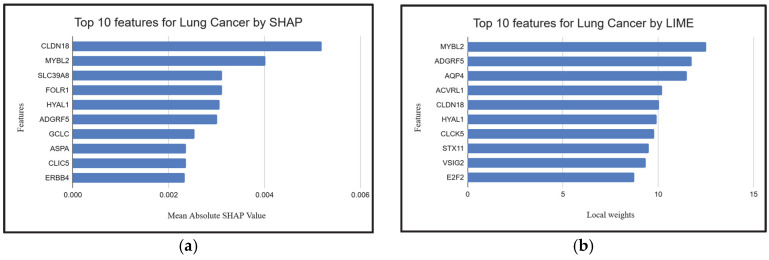
(**a**,**b**). Top 10 features for lung cancer given by SHAP and LIME.

**Figure 6:**
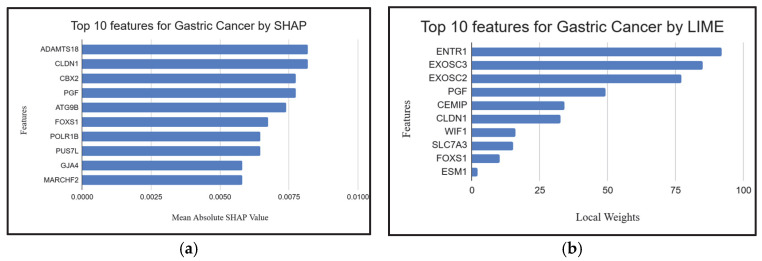
(**a**,**b**). Top 10 features for gastric cancer given by SHAP and LIME.

**Figure 7:**
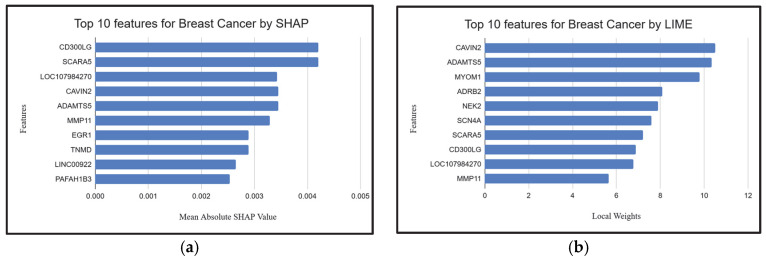
(**a**,**b**). Top 10 features for breast cancer given by SHAP and LIME.

To further interpret model predictions and validate biologically relevant features, we applied SHAP (Shapley Additive exPlanations) [87] and LIME (Local Interpretable Model-Agnostic Explanations) [87] to identify the top biomarker genes across lung, gastric, and breast cancer datasets. Both methods indicated high consistency with genes identified by XGB-BIF (the proposed methodology), supporting the robustness of our feature selection. For lung cancer, all manuscript-referenced genes were captured by either SHAP or LIME, with notable overlaps such as *MYBL2*, *HYAL1*, *CLIC5*, *ADGRF5*, and *CLDN18* (Figure 5a,b). In the gastric cancer dataset, three genes—*FOXS1*, *PGF*, and *CLDN1* (Figure 6a,b)—were commonly identified by both explainability methods and the manuscript, further validating their relevance. A strong convergence was also observed for breast cancer: all manuscript-listed genes, including *SCARA5*, *ADAMTS5*, *MMP11* and *CAVIN2* (Figure 7a,b), appeared in the outputs of SHAP or LIME. These results reinforce theiologyical validity of the proposed model and demonstrate the utility of XAI techniques in uncovering interpretable and credible genomic markers for cancer classification.

### 2.5. Results Obtained from the Survival Analysis

**Figure 8:**
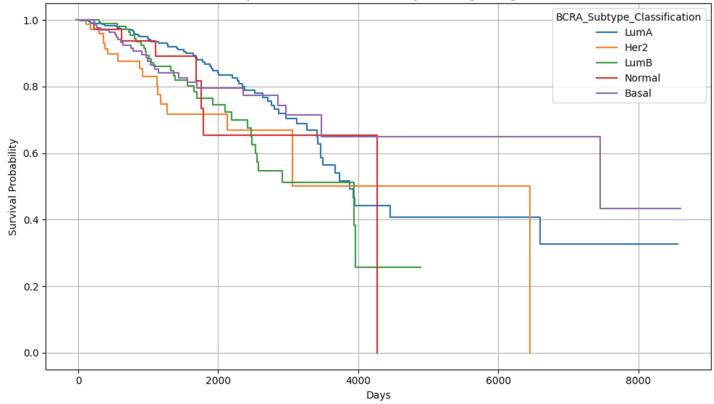
Kaplan–Meier curve by BRCA subtype.

In the current work, we performed survival analysis on the breast cancer data due to the easy availability of clinical metadata with the Kaplan–Meier survival curve, stratified by BRCA (breast cancer) subtype classification (Figure 8). The *x*-axis represents the number of days from diagnosis or study enrollment to either death or last follow-up. The *y*-axis represents the probability that a patient will survive past a certain number of days. LumA (blue line) tends to stay higher, which is consistent with better survival. LumB has steeper drops, suggesting worse survival. Basal also suggests a poor prognosis.

**Figure 9:**
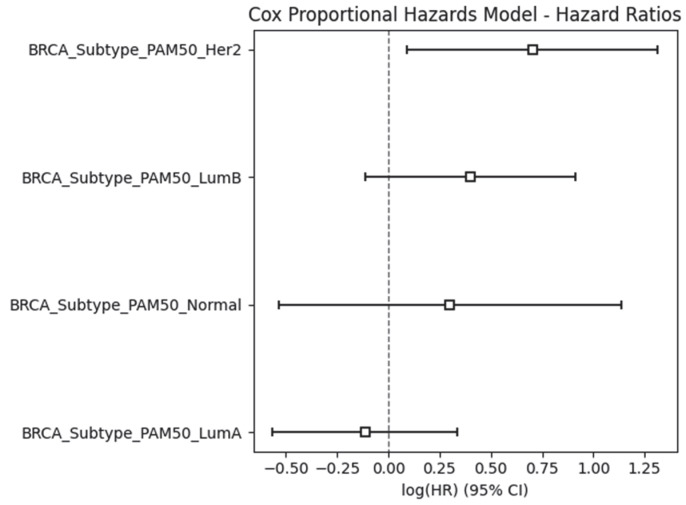
Applying Cox proportional hazards model to estimate the hazard ratios for each subtype.

To assess the prognostic importance of breast cancer molecular subtypes on patient survival, we additionally performed a multivariate Cox proportional hazards regression analysis using the lifelines 0.27.4 binary compatible with Python 3.9 package (accessed 30 May 2025). The analysis included the PAM50 BRCA subtypes (LumA, LumB, HER2-enriched, Basal-like, and Normal-like) as categorical predictors, with LumA serving as the reference category. Survival time was taken as the number of days from diagnosis to death or last follow-up, and event status was coded as 1 for deceased patients and 0 for censored individuals. The model revealed significant differences in hazard ratios among subtypes. Compared to Luminal A, the Basal-like and HER2-enriched subtypes were associated with higher hazard ratios, indicating poorer survival outcomes, while the Normal-like subtype showed variable results. These findings underscore the clinical relevance of BRCA subtype classification in the prognostic stratification of breast cancer patients (Figure 9). *p*-value < 0.05 suggests significant differences in survival between subtypes. If a CI crosses the dashed line at 0, the result is not statistically significant (*p*-value > 0.05). On the contrary, if the entire CI is on one side of 0, the difference is statistically significant. Her2 and LumB depict the worst prognosis, but LumA indicates possibly better survival.

## 3. Discussion

The proposed framework of ensemble methods is successfully used in biomarker identification and cancer prediction across all three datasets. There exists an association between the understanding of the diseased state (cancer) and the human genome. The current research continued constructing a computational model for linking the human genome by feature modeling within the popular XGB classifier, enabling a novel framework for supervised analysis.

To mitigate the effects of class imbalance between tumor and normal samples, we applied stratified sampling during cross-validation to ensure representative class distribution across all folds. Additionally, class weights were adjusted during model training to penalize the misclassification of minority class samples with ‘class_weight = ‘balanced’. These strategies aimed to enhance model fairness and performance across both classes.

In our current research, normalization and batch effect correction are critical preprocessing steps. While normalization (log transformation) adjusts for differences in library size and sequencing depth within samples, it does not eliminate systematic biases due to sampling in different labs, platforms, or processing times. To address this, we applied batch correction using the ComBat [88] algorithm, which adjusts both the mean and variance across batches while ComBat preserving the biological signal in TCGA datasets due to imbalanced phenotypes.

**Figure 10:**
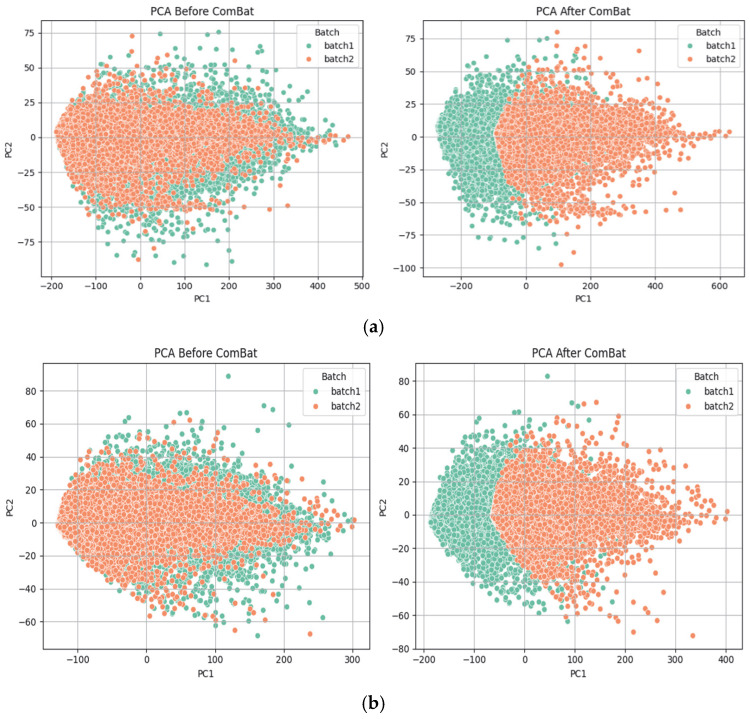
(**a**). Principal component analysis (PCA) before and after using ComBat on the over TCGA breast cancer dataset. (**b**). Principal component analysis (PCA) before and after using ComBat on the TCGA lung cancer dataset.

Figure 10 indicates that successfully removes the batch effect, aligning samples across batches while preserving the overall structure and aiding in downstream analysis.

Further in this research, the construction of an ensemble of XGB (feature selector) with meta-learners of SVM, RF, and LR over human genomes with two different phenotypes of tumorous case or non-tumorous has been undertaken. The accuracy of traditional RF, LR, and SVM is improved by integrating XGB-driven features (biomarkers) knowledge by minimizing the correlation between them and maximizing the predictive ability. XGB was especially selected because of its ability to handle high-dimensional data efficiently. XGB’s feature importance scores can be leveraged for effective feature selection, allowing researchers to attain the most relevant features and potentially enhance model performance. This aspect makes it suitable for gene expression datasets. XGB offers built-in feature importance scores and supports regularization to prevent overfitting. Importance calculated by XGB provides a score that indicates how useful each gene feature is in the construction of the boosted decision trees within the model. The more an attribute is used to make key decisions with decision trees, the higher its relative importance. The feature importance is averaged across all of the boosted decision trees within the mode. It is reputed to deliver superior predictive performance in classification tasks. The performance attained on top-ranked features (selected by XGB) was significantly better than the application of meta-learners on > 30,000 genes across their datasets. These qualities made XGB a viable choice for robust and scalable feature selection in our pipeline. To validate our pipeline, we also leveraged various feature selection methods and identified the features that were being selected as predictors for distinguishing tumors from normal tissue. These findings provide us valuable insights into the molecular mechanisms of the various cancers on which we worked. It also highlights potential biomarkers for diagnostic and therapeutic applications for the same.

Pathway enrichment analysis has revealed the significant activation of multiple oncogenic pathways across gastric cancer, breast cancer, and lung cancer datasets. Major signaling pathways, such as PI3K-AKT, Wnt/β-catenin, and p53, were universally enriched, highlighting their coordinating roles in tumorigenesis. Certain other genes, such as *HER2* (*ERBB2*) and *TP53* genes, were highly upregulated in gastric cancer, supporting their previously implicated roles in tumor growth, whereas *GKN1*, a tumor suppressor gene, was downregulated significantly. In the same way, in breast cancer, pathway analysis emphasized the role of extracellular matrix disassembly, hormone metabolism, and angiogenesis, where the major players were genes such as *MMP11* (facilitating metastasis) and *EGR1* (tumor suppressor). For lung cancer, major pathways included mitotic regulation, metabolic reprogramming, and tight junction integrity, having highly enriched genes such as *MYBL2* (driving cell cycle) and *FOLR1* (facilitating tumor growth through folate metabolism). Notably, genes such as *CLDN1* and *CLDN18* control cell adhesion and tight junctions and were found in both gastric and lung cancer. This indicates common mechanisms for tumor invasion and metastasis for both gastric as well as lung cancer. Ensemble learning techniques also served to increase biomarker discovery by combining several models to predict more accurately. These results aligned significantly with previous research and provided deeper insights into the molecular mechanisms driving these cancers. Several of the top-ranked genes identified by SHAP and LIME, explainable AI models (as indicated in the results section), overlapped with those identified in XGB-BIF to study cancer prognosis, thereby supporting the biological plausibility of our model. Our method was able to efficiently classify tumor and normal samples, showing strong performance across various datasets. The identification of new biomarker candidates proposes some directions for further investigation in the field of personalized cancer diagnosis and targeted treatments. Bulk RNA-seq data usage does not consider intratumorally heterogeneity, which might be resolved in the future using single-cell RNA-seq or spatial transcriptomics.

Moreover, although our ensemble approaches enhance the accuracy of prediction, experimental confirmation is required to validate the functional significance of identified biomarkers. In summary, our work establishes the power of ensemble-based gastric cancer, breast cancer, and lung cancer biomarker discovery and highlights the promise of such approaches for cancer research. Large-scale follow-up studies including multiple patient cohorts and multi-omics integration will be needed to confirm these observations and translate them into the clinical reality. Also, while performing the survival analysis of breast cancer, models revealed elevated hazard ratios for the *HER2*-enriched and LumB subtypes, suggesting poorer survival relative to the reference group, and LumA exhibited a trend toward better survival outcomes.

## 4. Materials and Methods

### 4.1. Materials

The study utilized high-throughput sequencing (HTS) data from RNA-based expression profiling to analyze metagenomes in gastric cancer. Additionally, breast invasive carcinoma (BRCA) and lung squamous cell carcinoma (LUSC) gene expression data were retrieved from the TCGA database (https://portal.gdc.cancer.gov accessed on 30 May 2025) using TCGA biolinks in R. The datasets are sourced as:

I. Gastric Cancer:

*GSE184336*: Transcriptome data from 231 gastric tumors and 230 paired normal gastric tissues, provided by Xue Y (SRP337610, RNA-seq, GSE184336) [89].

II. Breast Cancer:

*GSE62944:* Transcriptomic data from the TCGA-BRCA project were selected, comprising 1224 samples: 1111 primary tumor samples with varied subtypes and 113 normal solid tissue samples.

III. Lung Cancer:

*GSE62944:* Transcriptomic data from the TCGA-LUSC project were utilized, encompassing 562 samples—511 derived from primary tumors and 51 from normal solid tissues.

The focus was on distinguishing between ‘tumor’ and ‘paired normal tissue’ samples (Table 8).

### 4.2. Methods

**Figure 11:**
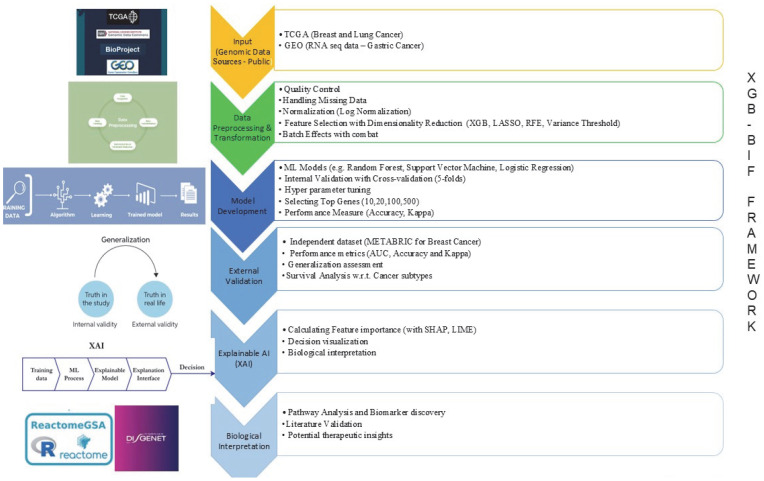
The proposed XGB-BIF framework.

In this research, we developed an innovative framework called XGB-BIF (Figure 11), which leverages XGB for the identification of biomarkers associated with cancer using human genetic data. The complexity and high dimensionality of genetic datasets necessitate effective feature selection methods, and our framework utilizes XGB capabilities to evaluate the importance of each gene or biomarker during model training. This section outlines the methodology, implementation, and evaluation of the XGB-BIF framework, highlighting its effectiveness in detecting relevant biomarkers for cancer.

Genetic datasets are high-dimensional, noisy, and complex, making feature selection crucial. XGB is used as a powerful feature selector in our framework due to its ability to evaluate the importance of each feature (gene/biomarker) during model training. XGB works by building decision trees, and during training, it tracks how useful each feature is at reducing loss. XGB also captures non-linear relationships and feature interactions better than simpler ML models like linear regression. Even with thousands of features (genes), XGB could rank them by relevance in an efficient way. XGB handles missing values internally and is less sensitive to outliers, so its importance scores were more stable than those of models like LR, SVM, and decision trees. This helped to identify the most relevant features, remove irrelevant or redundant features, speed up training time, and improve model generalization. Genetic datasets often have redundant or highly correlated features. XGB aided in pruning irrelevant features and focused on biologically meaningful markers. Additionally, XGB scaled efficiently on large genomic datasets. The code and datasets are available as Supplementary Material.

#### 4.2.1. Data Collection and Preprocessing

To ensure data quality and consistency, the following preprocessing steps were performed:

Gene Expression Data Processing:Transcriptomic data for breast and lung cancer were retrieved from the TCGA using the TCGA biolinks package. The original dataset comprised 60,660 ensemble gene identifiers. These were mapped to their corresponding gene names, resulting in 36,215 uniquely named genes. Genes with no expression across any of the samples were excluded, reducing the total to 35,083. To improve data quality and informativeness, genes in the lowest 10% variance range were also removed, resulting in 31,505 genes retained for downstream analysis and machine learning. Since normalization had already been completed, a log transformation was applied to the dataset to further standardize the distribution.

The gastric cancer dataset comprised 462 samples and 58,736 genes, offering a comprehensive view of transcriptomic activity. In contrast to the TCGA datasets, no additional filtering or transformation was applied, as the expression values were already suitably formatted. Further preprocessing was avoided to preserve potentially meaningful biological signals. The dataset’s large sample size ensured sufficient statistical power for model training without requiring additional adjustments.

Additional Preprocessing Measures:

- Dealing with Missing Data: To keep the dataset clean and reliable, any genes or samples with too many missing expression values were removed based on set cutoffs. This helped reduce unnecessary noise without losing important information.
- Scaling Gene Expression Values: A log transformation was applied to the datasets to values to smooth out the data, making gene expression levels easier to compare and improving how well the models could learn from the data.
- Balancing Sample Groups: Since there were differences in the number of healthy and diseased samples, we used stratified splitting when dividing the data into training and test sets. This ensured both groups were fairly represented in each set.
- Clinical Data Integration: We also gathered clinical details for all samples. This not only helped in clearly separating disease and control groups but also allowed us to include useful phenotypic traits in our analysis.
- Batch effects were removed using the ComBat method implemented in Python (pycombat library), which adjusts for both mean and variance across batches while preserving biological variability.

#### 4.2.2. Feature Selection Using XGB

Considering its built-in feature importance mechanism, we chose XGB to identify the key features. The process followed these steps: firstly, an XGB model was trained using the complete set of features. Following training, feature importance scores were obtained and calculated based on the frequency with which each feature was utilized within the model. Features that ranked above a predefined threshold were selected. Now, the dataset was subsequently reduced to retain only these top-ranked features for use in the downstream classification tasks.

XGB is an ensemble learning technique that applies boosting. Boosting, on the other hand, is an iterative process where weak learners are sequentially improved by assigning more weight to misclassified instances. The objective function of XGB consists of a loss function and a regularization term.

To identify a minimal and robust set of predictive biomarkers for comparative analysis with XGB, we employed Least Absolute Shrinkage and Selection Operator (LASSO) regression for feature selection. LASSO introduces an L1 penalty to the regression model, effectively shrinking the coefficients of less informative features (genes) to zero, thereby enabling both feature selection and model regularization [28].

We also employed recursive feature elimination (RFE) [29], a supervised wrapper-based feature selection technique that recursively removes the least informative features based on model performance. RFE was applied to the normalized gene expression matrix using a wrapper around a classification model of RF and SVM, allowing for feature ranking and dimensionality reduction.

#### 4.2.3. Classification Models

Three machine learning classifiers were implemented and evaluated on the reduced feature set: SVM, LR, and RF.

- SVM

SVM represent a category of supervised learning algorithms that can be utilized for either classification or regression purposes. The core concept behind SVM is to identify a hyperplane that optimally distinguishes the various classes present in the training data. This is achieved by locating the hyperplane that maintains the largest margin, defined as the distance from the hyperplane to the nearest data points of each class. Once the hyperplane is established, new data can be classified by assessing which side of the hyperplane it lies on. SVM are especially beneficial when the dataset contains numerous features and/or features a clear margin for separation among the data points. It is effective when working with high-dimensional datasets. This makes it a strong choice for applications like genomic data analysis. Kernelized SVM is used for non-linearly separable data. The radial basis function (RBF) kernel SVM was tested to evaluate its performance on high-dimensional genomic data.

Parameters in kernelized SVM are:

The Kernel: The type of transformation and the type of data are taken into consideration when choosing the kernel. The Radial Basis Function Kernel (RBF) is the kernel by default.

Gamma: This parameter determines the extent to which a single training example’s influence extends during transformation, thereby influencing the degree to which the decision boundaries ultimately encircle input space points. Points further apart are regarded as similar if the gamma value is small. As a result, there are more grouped points and smoother decision boundaries—though perhaps less accuracy. Points get closer together at larger gamma values, which could lead to overfitting.

‘C’ parameter: This parameter regulates how much regularization is applied to the data. Large values of C indicate low regularization, which leads to an extremely good fit (possibly overfitting) in the training set. Lower C values indicate higher regularization, which makes the model more error-tolerant and could result in decreased accuracy.

- LR

LR, a baseline classifier, is used to assess model performance on a simple linear decision boundary. LR is a widely used linear classification algorithm that estimates the probability of a sample belonging to a particular class using the logistic (sigmoid) function given in Equation (1):

(1) $$\sigma (z)=\frac{1}{1+e^{-z}}$$

It is especially effective for binary classification problems and is a strong baseline due to its simplicity, interpretability, and efficiency. In this study, LR was applied to gene expression data to classify cancer types, modeling the log-odds of the outcome as a linear combination of input features (genes). Both L1 (Lasso) and L2 (Ridge) regularization techniques were employed to prevent overfitting, especially in the high-dimensional genomic dataset. These regularization methods control model complexity and enhance generalization, with L1 encouraging feature sparsity and L2 penalizing large coefficient values. L1 and L2 regularization techniques were applied to avoid overfitting.

- RF

RF is a well-known supervised learning method suited for both classification and regression. It uses several decision trees on different dataset subsets and averages them to increase the dataset’s predictive accuracy.” Rather than depending on a single decision tree, the RF predicts the outcome based on the majority vote of predictions from each tree.

With high dimensionality, large datasets can be handled by RF. It keeps the overfitting problem at bay and improves the model’s accuracy.

The most important of these parameters that we need to tweak while hyperparameter tuning are as follows:

- n_estimators: The number of decision trees in the RF. We explored values between 100 and 500 to find the right balance between accuracy and computational efficiency.
- max_depth: The number of splits that each decision tree is allowed to make. If the number of splits is too low, the model underfits the data, and if it is too high, the model overfits. In this study, the tree depth was tuned between 5 and 20, allowing the model to learn meaningful patterns without becoming overly complex.
- max_features: The number of columns that are shown to each decision tree. The specific features that are passed to each decision tree can vary between each decision tree.
- Bootstrap sampling: A bootstrapped model takes only a select subset of columns and rows to train each decision tree. This way, each tree sees a slightly different subset of the data, which adds diversity to the model and helps prevent overfitting. This sampling approach also allows the algorithm to perform a kind of built-in feature selection since each tree is trained on a different subset of features.

These parameter choices helped create a model that is both stable and accurate, well-suited for the complexity and high dimensionality of biological data.

#### 4.2.4. Model Training and Evaluation

- Cross-Validation

The 5-fold cross-validation was applied to all the models for training and validation to ensure robustness and generalization. In this approach, the dataset D is divided into k=5 equal-sized subsets $D_{1},D_{2},\ldots ,D_{5}$. For each fold i ∈{1,2,5}, the model is trained on $D\backslash D_{i}$ and evaluated on $D_{i}$, the held-out fold. The average performance across all folds is computed as given in Equation (2):

(2) $$CVscore=\frac{1}{k}\Sigma i=1^{k}Score_{i}$$

where $Score_{i}$ is the performance metric (e.g., accuracy, F1-score) on the $i^{th}$ fold.

- Performance Metrics

The models were evaluated based on the following:

1. Accuracy: Accuracy [90] is one of the most commonly used metrics for classification problems. It reflects how often the model’s predictions align with the true labels. In simple terms, it is the proportion of correct predictions out of all the predictions made, and it is calculated as in Equation (3):

(3) $$Accuracy=\frac{TP+TN}{TP+TN+FP+FN}$$

where

- TP (True Positives): Cases where the model correctly predicted the positive class
- TN (True Negatives): Cases where the model correctly predicted the negative class
- FP (False Positives): Cases where the model incorrectly predicted the positive class
- FN (False Negatives): Cases where the model incorrectly predicted the negative class

In this study, accuracy refers to the percentage of gene expression samples that the model correctly identified as belonging to their respective cancer types. It gives a clear sense of how well the model performed in assigning the right labels based on the data.

2. Kappa: Cohen’s Kappa [90] is a statistical measure used to evaluate the agreement between predicted and actual classifications, adjusting for agreement that could occur by chance. This is especially valuable in imbalanced classification problems, where relying on accuracy alone may be misleading. The Kappa statistic is defined as Equation (4):

(4) $$\kappa =\frac{p_{o}-p_{e}}{1-p_{e}}$$

where

$p_{o}$: Observed agreement (i.e., accuracy)

$p_{e}$: Expected agreement by chance

A Kappa value of 1 indicates perfect agreement, 0 indicates no better than chance, and negative values suggest agreement worse than random guessing. Since our dataset contains class imbalance, Cohen’s Kappa was used alongside accuracy to provide a more balanced and reliable assessment of model performance.

- Hyperparameter Optimization

Hyperparameters were optimized with varied model settings to achieve the best classification performance.

#### 4.2.5. External Validation

To better evaluate the XGB-BIF framework, we further applied our trained model on METABRICS data [30], in which the samples are based on clinicopathological features to align with the training cohort (e.g., molecular subtype, ER status, or tumor grade). Gene expression data were log-transformed and quantile-normalized to ensure compatibility with the training data. Predictive models (e.g., random forest, SVM, and XGBoost) trained on the discovery data were directly applied to the METABRIC dataset using shared features. Performance was evaluated using area under the ROC curve (AUC-ROC) [90], accuracy, and Kappa.

#### 4.2.6. Explainable AI Methods

LIME and SHAP [87] methodologies were used in this research to find important cancer (gastric, lung, and breast) biomarkers in the patient group. LIME was used to provide local explanations in the XGB model. SHAP was used to provide a broad explanation for the XGB-BIF prediction model. The SHAP and LIME-based analyses revealed the most influential features in the model’s decision-making process. SHAP assigns each feature an importance value for a particular prediction based on its marginal contribution across all possible feature combinations. SHAP values were computed using the tree explainer method like XGBoost, LightGBM, etc., efficiently estimating feature attributions. We analyzed the distribution and magnitude of SHAP values to assess their impact on model output. On the other hand, LIME approximated complex model predictions by fitting an interpretable surrogate model, like the sparse linear model, locally around the predictions. LIME can effectively determine how much each gene in the data contributes to each (patient-specific) disease prediction in the model. Using the LIME method, it can be determined which biomarkers affect each prediction.

#### 4.2.7. Methods for Survival Analysis

To investigate the association between genomic features and patient survival outcomes, we conducted survival analysis using gene expression profiles of the breast cancer dataset. Clinical survival data, including overall survival time and event status (death or censoring), were integrated with normalized gene expression. We employed the Cox proportional hazards model [91] to estimate the relationship between gene expression levels and survival risk, and evaluated model performance using the concordance index (C-index), which reflects the model’s ability to correctly rank patients by risk. Patients were further stratified into subtypes of breast cancer, and Kaplan–Meier survival curves [89] were generated to visualize survival distributions. This aided in identifying gene expression signatures predictive of patient prognosis, highlighting potential biomarkers for risk stratification in TCGA breast cancer data.

#### 4.2.8. Software and Tools

- Programming Language: Python 3.9, R 4.4.0
- Libraries: sci-kit-learn, XGBoost, imbalanced-learn, NumPy, pandas, TCGABiolinks, SummarizedExperiment, dplyr, AnnotationDbi, org.Hs.eg.db, tidyverse
- The code and the data are available at https://github.com/MaitreyiComputationalBiology/XGB-BIF accessed on 3 June 2025.

#### 4.2.9. Biological Relevance Assessment

- To validate the biological significance of the selected features, pathway analysis was conducted using publicly available databases such as KEGG (Kyoto Encyclopedia of Genes and Genomes) and Reactome. The identified genes were mapped to known biological pathways to assess their functional roles in disease mechanisms. Additionally, a comprehensive literature review was performed using databases such as PubMed and Google Scholar to cross-reference the selected genetic markers with previously published studies. This helped in confirming whether the selected genes had been previously associated with the studied condition, ensuring the robustness of our feature selection in XGB-BIF.

Pathway enrichment analysis (PEA) serves as a crucial tool for validating biomarker discoveries by linking identified biomarkers to biological pathways, thereby providing context and functional relevance. This enhances the understanding of the underlying biological mechanisms associated with diseases, facilitating the identification of potential therapeutic targets. The following steps were undertaken for PEA:

- Given XGB’s outstanding predictive performance, we utilized its feature importance ranking to identify the top 10 most influential biomarkers (genes).
- Gene Ontology (GO) enrichment analysis was conducted using the enrichGO() function from the clusterProfiler package, specifically focusing on the Biological Process (BP) category, with an adjusted *p*-value threshold of 0.05.
- GO terms were filtered to retain only the most statistically significant pathways associated with each gene, ensuring biological relevance. Each gene was then annotated based on its established role in breast, gastric, and lung cancer, with supporting evidence gathered from literature and genomic databases.

## 5. Conclusions

Advances in genome technology have led to a new understanding of cancer and novel ways of diagnosing and treating many types of cancer. In our study, we have developed a framework for predicting gastric cancer, lung cancer, and breast cancer from gene sequencing data. Our framework XGB-BIF combines XGB as a feature selector alongside classifiers like LR, SVM, and RF for effective biomarker discovery and cancer classification. Our approach demonstrated high accuracy in predictions and consistency across gastric, breast, and lung cancer data sets, proving that XGB-based feature selection is effective for managing complex gene expression data. The identified biomarkers and notable cancer-related pathways provide important insights into how tumors grow, with specific genes, such as *HER2*, *TP53*, *MMP11*, *MYBL2*, and members of the *CLDN* family, showing strong connections to cancer progression. Pathway enrichment analyses further highlighted the activation of key signaling pathways—including PI3K-AKT, Wnt/β-catenin, and p53—showing their roles in tumor development across different types of cancer.

The proposed framework would be instrumental in combining various genomics data and advanced machine learning methods for personalized cancer diagnosis and targeted treatments.

## Figures and Tables

**Table 1:** Ensemble performance for gastric cancer vs. none feature ranking (Best performance is highlighted in Bold).

Feature Ranking	Classifier	Settings	Accuracy	Kappa
None	LR	Default	0.8817	0.7636
None	RF	Default	0.9355	0.8712
None	SVM	Default	0.8387	0.6781
XGB	LR	Top 500 features	0.9247	0.8496
**XGB**	**RF**	**Top 500 features**	**0.9462**	**0.8924**
XGB	SVM	Top 500 features	0.9354	0.8710
LASSO	RF	Default	0.9234	0.8312
LASSO	SVM	Default	0.9125	0.8516
RFE	RF	RFE with estimator RF	0.9218	0.8200
RFE	SVM	RFE with an estimator SVM	0.9213	0.8010
Variance Threshold	RF	Default with threshold value 0.05	0.9351	0.8700

**Table 2:** Ensemble performance for breast cancer vs. none feature ranking (Best performances are highlighted in Bold).

Feature Ranking	Classifier	Settings	Accuracy	Kappa
None	LR	Default	0.9864	0.9212
None	RF	Default	0.9755	0.8482
None	SVM	Default	0.9764	0.9079
**XGB**	**LR**	**Top 500 features**	**0.9918**	**0.9532**
XGB	RF	Top 500 features	0.9837	0.9201
**XGB**	**SVM**	**Top 500 features**	**0.9864**	**0.9179**
LASSO	RF	Default	0.9861	0.9168
LASSO	SVM	Default	0.9723	0.9126
RFE	RF	RFE with estimator RF	0.9771	0.8902
RFE	SVM	RFE with an estimator SVM	0.9674	0.9072
Variance threshold	RF	Default with threshold value 0.05	0.9882	0.92684

**Table 3:** Ensemble performance for lung cancer vs. none feature ranking (Best performances are highlighted in Bold).

Feature Ranking	Classifier	Settings	Accuracy	Kappa
None	LR	Default	0.9705	0.9412
None	RF	Default	0.9710	0.9405
None	SVM	Default	0.9712	0.9423
XGB	LR	Top 500 features	0.9882	0.9509
**XGB**	**RF**	**Top 500 features**	**0.9822**	**0.9868**
**XGB**	**SVM**	**Top 500 features**	**0.9941**	**0.9645**
LASSO	RF	Default	0.9861	0.9568
LASSO	SVM	Default	0.9723	0.9626
RFE	RF	RFE with estimator RF	0.9771	0.9432
RFE	SVM	RFE with an estimator SVM	0.9712	0.9412
Variance Threshold	RF	Default with threshold value 0.05	0.9687	0.9126

**Table 4:** External validation performance on the METABRIC dataset (Best performances are highlighted in Bold).

Feature Ranking	Classifier	Settings	AUC-ROC	Accuracy	Kappa
None	LR	Default	0.908	0.722	0.665
None	RF	Default	0.890	0.747	0.701
None	SVM	Default	0.920	0.723	0.721
XGB	LR	Top 500 features	0.924	0.750	0.690
**XGB**	**RF**	**Top 500 features**	0.924	0.782	0.721
**XGB**	**SVM**	**Top 500 features**	**0.935**	**0.786**	**0.740**

**Table 5:** Pathway enrichment analysis performed on top-identified genes in gastric cancer datasets.

EntrezID	Gene	Pathway	Role in Gastric Cancer
84733	*CBX2*	Polycomb repressive complex	Epigenetic silencing of tumor suppressor genes in gastric cancer [22].
9076	*CLDN1*	Cell adhesion molecules	Regulates gastric tumor cell adhesion, invasion, and metastasis [31].
		Virion-Hepatitis viruses	Hepatitis viruses may contribute to gastric cancer via chronic inflammation [32].
		Leukocyte transendothelial migration	Regulates immune infiltration in gastric tumor microenvironment [33].
		Hepatitis C	Chronic infection may promote gastric carcinogenesis [34].
		Tight junction	Dysfunction promotes gastric cancer cell invasion and metastasis [35].
		Pathogenic Escherichia coli infection	Chronic infection may alter gastric epithelial integrity [36].
5228	*PGF*	Focal adhesion	Mediates gastric cancer cell migration and invasion [37].
		Rap1 signaling pathway	Regulates gastric cancer cell adhesion and proliferation [38].
		Ras signaling pathway	Frequently mutated in gastric cancer, promoting cell proliferation [39].
		MAPK signaling pathway	Key driver of gastric cancer proliferation and survival [40].
		PI3K-Akt signaling pathway	Promotes gastric cancer growth and resistance to apoptosis [41].
8767	*RIPK2*	Neurotrophin signaling pathway	Involved in gastric cancer cell survival and proliferation [42].
		Tuberculosis	Chronic inflammation may contribute to gastric tumorigenesis [43].
		NOD-like receptor signaling pathway	Regulates inflammation-associated gastric carcinogenesis [44].
		Shigellosis	Infection-induced inflammation linked to gastric cancer risk [45].
		Salmonella infection	May contribute to gastric carcinogenesis via inflammation [46].
6383	*SDC2*	Cell adhesion molecules	Regulates gastric tumor cell adhesion, invasion, and metastasis [47].
		Malaria	Endothelial dysfunction linked to gastric disease progression [48].
		Fluid shear stress and atherosclerosis	Gastric cancer angiogenesis and microenvironment remodeling [49].
		Proteoglycans in cancer	Involved in gastric tumor progression and microenvironment interactions [50].

*CBX2* and *CLDN1* have been implicated in chromatin remodeling and cell adhesion processes critical to gastric cancer progression [51]. *PGF* is known to promote angiogenesis in tumors, while *RIPK2* contributes to inflammation-mediated tumorigenesis [52]. *SDC2* is a well-studied methylation biomarker in colorectal cancer [53].

**Table 8:** Summarized datasets.

Datasets	Genes	Samples	No. of Classes	Categories
Gastric cancer	58736	461	2	Gastric tumor (231) Paired normal tissue (230)
Breast cancer	31576	1224	2	Primary tumor (1111) Normal solid tissue (113)
Lung cancer	31507	562	2	Primary tumor (511) Normal solid tissue (51)

## Data Availability

No new data was created. Code and data is available at https://github.com/MaitreyiComputationalBiology/XGB-BIF, accessed on 3 June 2025.

## Supplementary Materials

The following supporting information can be downloaded at: https://www.mdpi.com/article/10.3390/ijms26125590/s1.

## References

[1] D’Argenio V., Dittfeld L., Lazzeri P., Tomaiuolo R., Tasciotti E. (2021). Unraveling the Balance between Genes, Microbes, Lifestyle and the Environment to Improve Healthy Reproduction. Genes.

[2] Weischenfeldt J., Symmons O., Spitz F., Korbel J.O. (2013). Phenotypic impact of genomic structural variation: Insights from and for human disease. Nat. Rev. Genet..

[3] Feuk L., Carson A.R., Scherer S.W. (2006). Structural variation in the human genome. Nat. Rev. Genet..

[4] Cao W., Chen H.-D., Yu Y.-W., Li N., Chen W.-Q. (2021). Changing profiles of cancer burden worldwide and in China: A secondary analysis of the global cancer statistics 2020. Chin. Med. J..

[5] Sulkowska U., Mańczuk M., Łobaszewski J., Zatoński W.A. (2015). Lung cancer, the leading cause of cancer deaths among women in Europe. Nowotwory. J. Oncol..

[6] Yang W.-J., Zhao H.P., Yu Y., Wang J.H., Guo L., Liu J.Y., Pu J., Lv J. (2023). Updates on global epidemiology, risk and prognostic factors of gastric cancer. World J. Gastroenterol..

[7] Tao Z., Shi A., Lu C., Song T., Zhang Z., Zhao J. (2015). Breast Cancer: Epidemiology and Etiology. Cell Biochem. Biophys..

[8] Rattray N.J.W., Charkoftaki G., Rattray Z., Hansen J.E., Vasiliou V., Johnson C.H. (2017). Environmental Influences in the Etiology of Colorectal Cancer: The Premise of Metabolomics. Curr. Pharmacol. Rep..

[9] Boyault S., Drouet Y., Navarro C., Bachelot T., Lasset C., Treilleux I., Tabone E., Puisieux A., Wang Q. (2012). Mutational characterization of individual breast tumors: TP53 and PI3K pathway genes are frequently and distinctively mutated in different subtypes. Breast Cancer Res. Treat..

[10] Testa U., Castelli G., Pelosi E. (2018). Lung Cancers: Molecular Characterization, Clonal Heterogeneity and Evolution, and Cancer Stem Cells. Cancers.

[11] Skoulidis F., Heymach J.V. (2019). Co-occurring genomic alterations in non-small-cell lung cancer biology and therapy. Nat. Rev. Cancer.

[12] Hayashi R., Inomata M. (2022). Small cell lung cancer; recent advances of its biology and therapeutic perspective. Respir. Investig..

[13] Wang R.C., Wang Z. (2023). Precision Medicine: Disease Subtyping and Tailored Treatment. Cancers.

[14] Weitzel J.N., Blazer K.R., MacDonald D.J., Culver J.O., Offit K. (2011). Genetics, genomics, and cancer risk assessment: State of the Art and Future Directions in the Era of Personalized Medicine. CA A Cancer J. Clin..

[15] Nagaraj A. (2023). COVID-19—Monitoring with IoT Devices.

[16] Wang Z., Gao X., Zeng R., Wu Q., Sun H., Wu W., Zhang X., Sun G., Yan B., Wu L. (2020). Changes of the Gastric Mucosal Microbiome Associated with Histological Stages of Gastric Carcinogenesis. Front. Microbiol..

[17] Sterbini F.P., Palladini A., Masucci L., Cannistraci C.V., Pastorino R., Ianiro G., Bugli F., Martini C., Ricciardi W., Gasbarrini A. (2016). Effects of Proton Pump Inhibitors on the Gastric Mucosa-Associated Microbiota in Dyspeptic Patients. Appl. Environ. Microbiol..

[18] Qi G.-J., Luo J. (2020). Small Data Challenges in Big Data Era: A Survey of Recent Progress on Unsupervised and Semi-Supervised Methods. IEEE Trans. Pattern Anal. Mach. Intell..

[19] You L., Dou Y., Zhang Y., Xiao H., Lv H., Wei G.H., Xu D. (2023). SDC2 Stabilization by USP14 Promotes Gastric Cancer Progression through Co-option of PDK1. Int. J. Biol. Sci..

[20] Huang J., Zhang L., He C., Qu Y., Li J., Zhang J., Du T., Chen X., Yu Y., Liu B. (2014). Claudin-1 enhances tumor proliferation and metastasis by regulating cell anoikis in gastric cancer. Oncotarget.

[21] Yang Y., Zufu J., Weizhou W., Libin R., Chengyang Y., Yuning X., Liling W., Kunpeng W., Jinggang M., Shankun Z. (2021). Chronic Hepatitis Virus Infection Are Associated with High Risk of Gastric Cancer: A Systematic Review and Cumulative Analysis. Front. Oncol..

[22] Ma R., Zhang Y., Sun T., Cheng B. (2014). Epigenetic regulation by polycomb group complexes: Focus on roles of CBX proteins. J. Zhejiang Univ. Sci. B.

[23] Morgos D.-T., Stefani C., Miricescu D., Greabu M., Stanciu S., Nica S., Stanescu S., Balan D.G., Balcangiu S., Coculescu E.C. (2024). Targeting PI3K/AKT/mTOR and MAPK Signaling Pathways in Gastric Cancer. Int. J. Mol. Sci..

[24] Wang M., Zhang C., Song Y., Wang Z., Wang Y., Luo F., Xu Y., Zhao Y., Wu Z., Xu Y. (2017). Mechanism of immune evasion in breast cancer. OncoTargets Ther..

[25] Gilmore E., McCabe N., Kennedy R.D., Parkes E.E. (2019). DNA Repair Deficiency in Breast Cancer: Opportunities for Immunotherapy. J. Oncol..

[26] Miller T.W. (2012). Initiating breast cancer by PIK3CA mutation. Breast Cancer Res..

[27] Theng D., Bhoyar K.K. (2024). Feature selection techniques for machine learning: A survey of more than two decades of research. Knowl. Inf. Syst..

[28] Muthukrishnan R., Rohini R. (2016). LASSO: A Feature Selection Technique in Predictive Modeling for Machine Learning. Proceedings of the 2016 IEEE International Conference on Advances in Computer Applications (ICACA).

[29] Rani P., Kumar R., Jain A., Chawla S.K. (2021). A Hybrid Approach for Feature Selection Based on Genetic Algorithm and Recursive Feature Elimination. Int. J. Inf. Syst. Model. Des..

[30] Curtis C., Shah S.P., Chin S.-F., Turashvili G., Rueda O.M., Dunning M.J., Speed D., Lynch A.G., Samarajiwa S., Yuan Y. (2012). The genomic and transcriptomic architecture of 2,000 breast tumours reveals novel subgroups. Nature.

[31] Eftang L.L., Esbensen Y., Tannæs T.M., Blom G.P., Bukholm I.R., Bukholm G. (2013). Up-regulation of CLDN1 in gastric cancer is correlated with reduced survival. BMC Cancer.

[32] Niedźwiedzka-Rystwej P., Grywalska E., Hrynkiewicz R., Wołącewicz M., Becht R., Roliński J. (2020). The Double-Edged Sword Role of Viruses in Gastric Cancer. Cancers.

[33] Sekhar V., Pollicino T., Diaz G., Engle R.E., Alayli F., Melis M., Kabat J., Tice A., Pomerenke A., Altan-Bonnet N. (2018). Infection with hepatitis C virus depends on TACSTD2, a regulator of claudin-1 and occludin highly downregulated in hepatocellular carcinoma. PLoS Pathog..

[34] Bhat A.A., Syed N., Therachiyil L., Nisar S., Hashem S., Macha M.A., Yadav S.K., Krishnankutty R., Muralitharan S., Al-Naemi H. (2020). Claudin-1, A Double-Edged Sword in Cancer. Int. J. Mol. Sci..

[35] Gong Y., Jin X., Yuan B., Lv Y., Yan G., Liu M., Xie C., Liu J., Tang Y., Gao H. (2021). G Protein-Coupled Receptor 109A Maintains the Intestinal Integrity and Protects Against ETEC Mucosal Infection by Promoting IgA Secretion. Front. Immunol..

[36] Menter D.G., DuBois R.N. (2012). Prostaglandins in Cancer Cell Adhesion, Migration, and Invasion. Int. J. Cell Biol..

[37] Lai I.-R., Chu P.Y., Lin H.S., Liou J.Y., Jan Y.J., Lee J.C., Shen T.L. (2010). Phosphorylation of Focal Adhesion Kinase at Tyr397 in Gastric Carcinomas and its Clinical Significance. Am. J. Pathol..

[38] Yang Y., Zhang J., Yan Y., Cai H., Li M., Sun K., Wang J., Liu X., Wang J., Duan X. (2017). Low expression of Rap1GAP is associated with epithelial-mesenchymal transition (EMT) and poor prognosis in gastric cancer. Oncotarget.

[39] Fujita K., Ohuchi N., Yao T., Okumura M., Fukushima Y., Kanakura Y., Kitamura Y., Fujita J. (1987). Frequent overexpression, but not activation by point mutation, of ras genes in primary human gastric cancers. Gastroenterology.

[40] Magnelli L., Schiavone N., Staderini F., Biagioni A., Papucci L. (2020). MAP Kinases Pathways in Gastric Cancer. Int. J. Mol. Sci..

[41] Matsuoka T., Yashiro M. (2014). The Role of PI3K/Akt/mTOR Signaling in Gastric Carcinoma. Cancers.

[42] Yang Q., Hong K., Li Y., Shi P., Yan F., Zhang P. (2024). Receptor-interacting protein kinase 2 is associated with tumor immune infiltration, immunotherapy-related biomarkers, and affects gastric cancer cells growth in vivo. J. Cancer.

[43] Yang Q., Tian S., Liu Z., Dong W. (2021). Knockdown of RIPK2 Inhibits Proliferation and Migration, and Induces Apoptosis via the NF-κB Signaling Pathway in Gastric Cancer. Front. Genet..

[44] Zhou Y., Yu S., Zhang W. (2023). NOD-like Receptor Signaling Pathway in Gastrointestinal Inflammatory Diseases and Cancers. Int. J. Mol. Sci..

[45] Philpott D.J., Sorbara M.T., Robertson S.J., Croitoru K., Girardin S.E. (2014). NOD proteins: Regulators of inflammation in health and disease. Nat. Rev. Immunol..

[46] Negroni A., Colantoni E., Cucchiara S., Stronati L. (2020). Necroptosis in Intestinal Inflammation and Cancer: New Concepts and Therapeutic Perspectives. Biomolecules.

[47] Liao S., Liu C., Zhu G., Wang K., Yang Y., Wang C. (2020). Relationship between SDC1 and cadherin signalling activation in cancer. Pathol.-Res. Pract..

[48] Wu S., Nie Q., Tan S., Liao G., Lv Y., Lv C., Chen G., Liu S. (2023). The immunity modulation of transforming growth factor-β in malaria and other pathological process. Int. Immunopharmacol..

[49] Zhang R., Song B., Hong X., Shen Z., Sui L., Wang S. (2020). microRNA-9 Inhibits Vulnerable Plaque Formation and Vascular Remodeling via Suppression of the SDC2-Dependent FAK/ERK Signaling Pathway in Mice with Atherosclerosis. Front. Physiol..

[50] De Pasquale V., Pavone L.M. (2020). Heparan Sulfate Proteoglycan Signaling in Tumor Microenvironment. Int. J. Mol. Sci..

[51] Hashimoto I., Oshima T. (2022). Claudins and Gastric Cancer: An Overview. Cancers.

[52] Zhou Y., Xiang Y., Liu S., Li C., Dong J., Kong X., Ji X., Cheng X., Zhang L. (2024). RIPK3 signaling and its role in regulated cell death and diseases. Cell Death Discov..

[53] Xu X., Guo Y., Liu M., Hu Y., Li S. (2024). Advancements in the clinical application of gene methylation for early cancer detection. Front. Epigenet. Epigenom..

[54] Souza M.C., Nunes S., Figuerêdo S.H.S., de Almeida B.S., Santos I.P.C., Cassali G.D., Arruda S.M., Cardoso T.M.S., Estrela-Lima A., Damasceno K.A. (2024). Versican Proteolysis by ADAMTS: Understanding Versikine Expression in Canine Spontaneous Mammary Carcinomas. Cancers.

[55] Mou K., Wang H., Zhu S., Luo J., Wang J., Peng L., Lei Y., Zhang Y., Huang S., Zhao H. (2024). Comprehensive analysis of the prognostic and immunological role of cavins in non-small cell lung cancer. BMC Cancer.

[56] Kang S.U., Cho S.Y., Jeong H., Han J., Chae H.Y., Yang H., Sung C.O., Choi Y.-L., Shin Y.K., Kwon M.J. (2022). Matrix Metalloproteinase 11 (MMP11) in Macrophages Promotes the Migration of HER2-Positive Breast Cancer Cells and Monocyte Recruitment through CCL2–CCR2 Signaling. Lab. Investig..

[57] You K., Su F., Liu L., Lv X., Zhang J., Zhang Y., Liu B. (2017). SCARA5 Plays a Critical Role in the Progression and Metastasis of Breast Cancer by Inactivating the ERK1/2, STAT3, and AKT Signaling Pathways. Mol. Cell. Biochem..

[58] Rasras S., Akade E., Mohammadianinejad S.E., Barahman M., Bahadoram M. (2024). Early growth response 1 transcription factor and its context-dependent functions in glioblastoma. Contemp. Oncol..

[59] Fontanil T., Álvarez-Teijeiro S., Villaronga M.Á., Mohamedi Y., Solares L., Moncada-Pazos A., Vega J.A., García-Suárez O., Pérez-Basterrechea M., García-Pedrero J.M. (2017). Cleavage of Fibulin-2 by the aggrecanases ADAMTS-4 and ADAMTS-5 contributes to the tumorigenic potential of breast cancer cells. Oncotarget.

[60] Han Q., Qiu S., Hu H., Li W., Li X. (2023). Role of Caveolae family-related proteins in the development of breast cancer. Front. Mol. Biosci..

[61] De Vega R.G., Clases D., Fernández-Sánchez M.L., Eiró N., González L.O., Vizoso F.J., Doble P.A., Sanz-Medel A. (2018). MMP-11 as a biomarker for metastatic breast cancer by immunohistochemical-assisted imaging mass spectrometry. Anal. Bioanal. Chem..

[62] (2025). SCARA5 Scavenger Receptor Class A Member 5. National Center of Biotechnology Information. https://www.ncbi.nlm.nih.gov/gene/?term=286133.

[63] Ni Q., Li X., Huang H., Ge Z. (2023). Decreased expression of SCARA5 predicts a poor prognosis in melanoma using bioinformatics analysis. Front. Oncol..

[64] Inoue K., Fry E.A. (2018). Tumor suppression by the EGR1, DMP1, ARF, p53, and PTEN Network. Cancer Investig..

[65] (2025). EGR1 Early Growth Response 1. National Center of Biotechnology Information. https://www.ncbi.nlm.nih.gov/gene?Cmd=DetailsSearch&Db=gene&Term=1958&utm_source=chatgpt.com.

[66] Hansson M.L., Behmer S., Ceder R., Mohammadi S., Preta G., Grafström R.C., Fadeel B., Wallberg A.E. (2012). MAML1 Acts Cooperatively with EGR1 to Activate EGR1-Regulated Promoters: Implications for Nephrogenesis and the Development of Renal Cancer. PLoS ONE.

[67] Ho L.-C., Sung J.M., Shen Y.T., Jheng H.F., Chen S.H., Tsai P.J., Tsai Y.S. (2016). Egr-1 deficiency protects from renal inflammation and fibrosis. J. Mol. Med..

[68] Xie H. (2020). The Epigenetic Role of EGR1 During Postnatal Brain Development and in Neuronal Activity. Ph.D. Thesis.

[69] Shajahan-Haq A.N., Boca S.M., Jin L., Bhuvaneshwar K., Gusev Y., Cheema A.K., Demas D.D., Raghavan K.S., Michalek R., Madhavan S. (2017). EGR1 regulates cellular metabolism and survival in endocrine resistant breast cancer. Oncotarget.

[70] Xie Y., Wen X., Jiang Z., Fu H.Q., Han H., Dai L. (2012). Aquaporin 1 and aquaporin 4 are involved in invasion of lung cancer cells. Clin. Lab..

[71] Hu Q., Li Y., Li D., Yuan Y., Wang K., Yao L., Cheng Z., Han T. (2023). Amino acid metabolism regulated by lncRNAs: The propellant behind cancer metabolic reprogramming. Cell Commun. Signal..

[72] Yang W., Qiu C., Biswas N., Jin J., Watkins S.C., Montelaro R.C., Coyne C.B., Wang T. (2008). Correlation of the Tight Junction-like Distribution of Claudin-1 to the Cellular Tropism of Hepatitis C Virus. J. Biol. Chem..

[73] Soini Y. (2012). Tight junctions in lung cancer and lung metastasis: A review. Int. J. Clin. Exp. Pathol..

[74] Li Q., Zhang Q., Wang C., Li N., Li J. (2008). Invasion of enteropathogenic *Escherichia coli* into host cells through epithelial tight junctions. FEBS J..

[75] Walter R.F.H., Walter R.F., Mairinger F.D., Werner R., Vollbrecht C., Hager T., Schmid K.W., Wohlschlaeger J., Christoph D.C. (2016). Folic-acid metabolism and DNA-repair phenotypes differ between neuroendocrine lung tumors and associate with aggressive subtypes, therapy resistance and outcome. Oncotarget.

[76] Han X., Chen L., Hu Z., Chen L., Sun P., Wang Y., Liu Y. (2021). Identification of proteins related with pemetrexed resistance by iTRAQ and PRM-based comparative proteomic analysis and exploration of IGF2BP2 and FOLR1 functions in non-small cell lung cancer cells. J. Proteom..

[77] Nawaz F.Z., Kipreos E.T. (2022). Emerging roles for folate receptor FOLR1 in signaling and cancer. Trends Endocrinol. Metab..

[78] Ahmed F. (2019). Integrated Network Analysis Reveals FOXM1 and MYBL2 as Key Regulators of Cell Proliferation in Non-small Cell Lung Cancer. Front. Oncol..

[79] Nebert D.W., Liu Z. (2019). SLC39A8 gene encoding a metal ion transporter: Discovery and bench to bedside. Hum. Genom..

[80] Li J., Yang D., Lin L., Yu L., Chen L., Lu K., Lan J., Zeng Y., Xu Y. (2024). Important functions and molecular mechanisms of aquaporins family on respiratory diseases: Potential translational values. J. Cancer.

[81] Lin G., Chen L., Lin L., Lin H., Guo Z., Xu Y., Hu C., Fu J., Lin Q., Chen W. (2021). Comprehensive Analysis of Aquaporin Superfamily in Lung Adenocarcinoma. Front. Mol. Biosci..

[82] Han Y., Wang X., Xu M., Teng Z., Qin R., Tan G., Li P., Sun P., Liu H., Chen L. (2023). Aspartoacylase promotes the process of tumour development and is associated with immune infiltrates in gastric cancer. BMC Cancer.

[83] Liu J., Yang H., Yin D., Jia Y., Li S., Liu Y. (2022). Expression and prognostic analysis of CLDN18 and Claudin18.2 in lung adenocarcinoma. Pathol.-Res. Pract..

[84] Varaganti P., Buddolla V., Lakshmi B.A., Kim Y.-J. (2023). Recent advances in using folate receptor 1 (FOLR1) for cancer diagnosis and treatment, with an emphasis on cancers that affect women. Life Sci..

[85] Lee Y., Wu Z., Yang S., Schreiner S.M., Gonzalez-Smith L.D., Rhie S.K. (2022). Characterizing and Targeting Genes Regulated by Transcription Factor MYBL2 in Lung Adenocarcinoma Cells. Cancers.

[86] Zhou H., Zhu Y., Qi H., Liang L., Wu H., Yuan J., Hu Q. (2021). Evaluation of the prognostic values of solute carrier (SLC) family 39 genes for patients with lung adenocarcinoma. Aging.

[87] Holzinger A., Saranti A., Molnar C., Biecek P., Samek W., Holzinger A., Goebel R., Fong R., Moon T., Müller K.-R., Samek W. (2022). Explainable AI Methods—A Brief Overview. xxAI-Beyond Explainable AI.

[88] Zhang Y., Parmigiani G., Johnson W.E. (2020). ComBat-seq: Batch effect adjustment for RNA-seq count data. NAR Genom. Bioinform..

[89] GEO Accession Viewer. https://www.ncbi.nlm.nih.gov/geo/query/acc.cgi?acc=GSE184336.

[90] Naidu G., Zuva T., Sibanda E.M., Silhavy R., Silhavy P. (2023). A Review of Evaluation Metrics in Machine Learning Algorithms. Artificial Intelligence Application in Networks and Systems, Proceedings of the 12th Computer Science Online Conference 2023, Online, 3–5 April 2023.

[91] Schober P., Vetter T.R. (2021). Kaplan-Meier curves, log-rank tests, and cox regression for time-to-event data. Anesth. Analg..

