# Supplementary material for: XGB-BIF: An XGBoost-Driven Biomarker Identification Framework for Detecting Cancer Using Human Genomic Data
Source: Int J Mol Sci. 2025 Jun 11;26(12):5590. doi: 10.3390/ijms26125590 (PMC12192969; doi:10.3390/ijms26125590)
Supplement: Supplementary file 1 [file ijms-26-05590-s001.zip › ijms-3613931-supplementary.pdf]

Supplementary Tables

Table A1. Feature selectors used for gastric cancer for comparative basis

Feature Selection	Classifier	Settings (Top n features)	Accuracy	Kappa
**RF**	**SVM**	Top 10 features	0.8925	0.7852
		Top 50 features	0.914	0.828
		Top 100 features	0.9247	0.8495
		Top 500 features	0.9247	0.8495
		Top 1000 features	0.914	0.8281
**RF**	**LR**	Top 10 features	0.892473	0.78512
		Top 50 features	0.946237	0.892486
		Top 100 features	0.935484	0.871012
		Top 500 features	0.935484	0.871012
		Top 1000 features	0.924731	0.849619
**RF**	**RF**	Top 10 features	0.946237	0.892486
		Top 50 features	0.935484	0.870953
		Top 100 features	0.935484	0.870953
		Top 500 features	0.956989	0.913969
**Variance Threshold**	**XGB**	0.001	0.935484	0.871012
		0.005	0.935484	0.871012
		0.01	0.946237	0.892486
		0.05	0.935484	0.871012
		0.1	0.946237	0.892486
**Variance Threshold**	**SVM**	0.001	0.913978	0.827937
		0.005	0.892473	0.785021

		0.01	0.88172	0.763578
		0.05	0.892473	0.78512
		0.1	0.892473	0.78512
**Variance Threshold**	**LR**	0.001	0.913978	0.828176
		0.005	0.903226	0.806742
		0.01	0.913978	0.828176
		0.05	0.924731	0.849549
		0.1	0.924731	0.849549
**Variance Threshold**	**RF**	0.001	0.935484	0.871012
		0.005	0.935484	0.871012
		0.01	0.913978	0.828017
		0.05	0.935484	0.871012
		0.1	0.935484	0.871012
**Mutual Information**	**SVM**	Top 10 features	0.903226	0.806653
		Top 50 features	0.913978	0.828096
		Top 100 features	0.913978	0.828096
		Top 500 features	0.913978	0.828096
		Top 1000 features	0.913978	0.828096
**Mutual Information**	**LR**	Top 10 features	0.892473	0.785219
		Top 50 features	0.946237	0.892486
		Top 100 features	0.946237	0.892486
		Top 500 features	0.903226	0.806653
		Top 1000 features	0.946237	0.892535
**Mutual Information**	**RF**	Top 10 features	0.946237	0.892486
		Top 50 features	0.935484	0.870953
		Top 100 features	0.935484	0.870953
		Top 500 features	0.946237	0.892486

		Top 1000 features	0.946237	0.892486

Table A2. Feature selectors used for breast cancer for comparative basis

Feature Selection	Classifier	Settings	Accuracy	Kappa
**RF**	**SVM**	Top 10 features	0.8925	0.7852
		Top 50 features	0.914	0.828
		Top 100 features	0.9247	0.8495
		Top 500 features	0.9247	0.8495
		Top 1000 features	0.914	0.8281
**RF**	**LR**	Top 10 features	0.892473	0.78512
		Top 50 features	0.946237	0.892486
		Top 100 features	0.935484	0.871012
		Top 500 features	0.935484	0.871012
		Top 1000 features	0.924731	0.849619
**RF**	**RF**	Top 10 features	0.946237	0.892486
		Top 50 features	0.935484	0.870953
		Top 100 features	0.935484	0.870953
		Top 500 features	0.956989	0.913969
		Top 1000 features	0.946237	0.892486
**Variance Threshold**	**SVM**	0.001	0.913978	0.827937
		0.005	0.892473	0.785021
		0.01	0.88172	0.763578
		0.05	0.892473	0.78512
		0.1	0.892473	0.78512
**Variance Threshold**	**LR**	0.001	0.913978	0.828176
		0.005	0.903226	0.806742

		0.01	0.913978	0.828176
		0.05	0.924731	0.849549
		0.1	0.924731	0.849549
**Variance Threshold**	**RF**	0.001	0.935484	0.871012
		0.005	0.935484	0.871012
		0.01	0.913978	0.828017
		0.05	0.935484	0.871012
		0.1	0.935484	0.871012
**Mutual Information**	**SVM**	Top 10 features	0.903226	0.806653
		Top 50 features	0.913978	0.828096
		Top 100 features	0.913978	0.828096
		Top 500 features	0.913978	0.828096
		Top 1000 features	0.913978	0.828096
**Mutual Information**	**LR**	Top 10 features	0.892473	0.785219
		Top 50 features	0.946237	0.892486
		Top 100 features	0.946237	0.892486
		Top 500 features	0.903226	0.806653
		Top 1000 features	0.946237	0.892535
**Mutual Information**	**RF**	Top 10 features	0.946237	0.892486
		Top 50 features	0.935484	0.870953
		Top 100 features	0.935484	0.870953
		Top 500 features	0.946237	0.892486
		Top 1000 features	0.946237	0.892486

Table A3. Feature selectors used for lung cancer for comparative basis

Feature Selection	Classifier	Settings	Accuracy	Kappa
**RF**	**SVM**	Top 10 features	0.9882	0.9268

		Top 50 features	0.9841	0.9445
		Top 100 features	0.9841	0.9545
		Top 500 features	0.9841	0.9545
**RF**	**LR**	Top 10 features	0.982249	0.893465
		Top 50 features	0.988166	0.930992
		Top 100 features	0.988166	0.930992
		Top 500 features	0.988166	0.930992
		Top 1000 features	0.982249	0.899345
**RF**	**RF**	Top 10 features	0.96166	0.92684
		Top 50 features	0.96166	0.92684
		Top 100 features	0.96166	0.92684
		Top 500 features	0.96816	0.92684
**Variance Threshold**	**SVM**	0.001	0.9984083	0.944488
		0.005	0.984083	0.944488
		0.01	0.984083	0.944488
		0.05	0.974083	0.956488
		0.1	0.984083	0.956488
**Variance Threshold**	**LR**	0.001	0.934911	0.697674
		0.005	0.934911	0.697674
		0.01	0.934911	0.697674
		0.05	0.934911	0.697674
		0.1	0.934911	0.697674
**Variance Threshold**	**RF**	0.001	0.974083	0.954488
		0.005	0.974083	0.954488
		0.01	0.988166	0.92684
		0.05	0.974083	0.954488
		0.1	0.94083	0.954488

**Mutual Information**	**SVM**	Top 10 features	0.988166	0.92684
		Top 50 features	0.984083	0.954488
		Top 100 features	0.988166	0.92684
		Top 500 features	0.984083	0.954488
		Top 1000 features	0.974083	0.943488
**Mutual Information**	**LR**	Top 10 features	0.977146	0.92684
		Top 50 features	0.977166	0.920992
		Top 100 features	0.977166	0.920992
		Top 500 features	0.987166	0.920992
		Top 1000 features	0.988166	0.920992
**Mutual Information**	**RF**	Top 10 features	0.972249	0.886856
		Top 50 features	0.978166	0.91684
		Top 100 features	0.978166	0.91684
		Top 500 features	0.978166	0.91684
		Top 1000 features	0.978166	0.92784
